# Influence of pH on the Formation of Benzyl Ester Bonds Between Dehydrogenation Polymers and Konjac Glucomannan

**DOI:** 10.3390/molecules29215166

**Published:** 2024-10-31

**Authors:** Peng Wang, Xu Zhang, Xi Le, Junjun Chen, Guangyan Zhang, Junjian An, Nianjie Feng, Junxian Xie

**Affiliations:** 1 Hubei Provincial Key Laboratory of Green Materials for Light Industry, Hubei University of Technology, Wuhan 430068, China; 2 School of Materials and Chemical Engineering, Hubei University of Technology, Wuhan 430068, China

**Keywords:** dehydrogenation polymer, konjac glucomannan, quinone methides, benzyl ester bond

## Abstract

A thorough understanding of the lignin–carbohydrate complex (LCC) structure has a significant meaning in the high-value utilization of lignocellulose. In this work, the complex (DHPKGC) was obtained by an addition reaction between konjac glucomannan (KGM) and quinone methides generated in the synthesis of dehydrogenation polymers (DHPs) to simulate the formation of LCCs. The effect of pH on the prepared DHPKGC was investigated. The structure of the DHPKGC was characterized by Fourier Transform Infrared (FTIR), ^13^C-Nuclear Magnetic Resonance (^13^C-NMR), and two-dimensional Heteronuclear Single Quantum Coherence Nuclear Magnetic Resonance (2D HSQC NMR) analyses. The results indicated the pH of 4.0 was conducive to the polymerization reaction between DHPs and oxidized KGM by the TEMPO/NaClO/NaBr system. In addition, the resultant DHPKGC was connected by benzyl ester linkages. Overall, this study aims to gain greater insight into the process of LCC formation in plants.

## 1. Introduction

The plant cell wall is mainly composed of cellulose, hemicellulose, and lignin [1]. The principal roles of lignin in plants include providing physical strength and water resistance and protecting plants from microorganisms and insects [2]. During lignin biosynthesis, two phenoxyl radicals are coupled to generate quinone methide [3]. The addition reaction of carbohydrates with the quinone methide generates the lignin–carbohydrate complex (LCC) [4,5,6]. The LCC is of great significance in both the mechanical resilience of plant cell walls and the potential for transformation such as pulp or chemical production, biodegradation, and other applications [7,8,9]. Thus, grasping the formation process and its interactions between lignin and polysaccharides contributes to the understanding of cell wall structure, which can also be used to synthesize LCC-based biomaterials [10,11,12].

Numerous experiments on LCCs strongly suggested the existence of covalent bond connections between lignin and hemicellulose in plants [5,13]. Hemicellulose is not a homogeneous glycan but a general term for a group of complex glycans. The main structural units that make up hemicellulose are monosaccharides (e.g., xylose, glucose, and mannose) and uronic acid (e.g., glucuronic acid) [14]. The addition reaction of the carboxyl group of the uronic acid in hemicellulose with the quinone methide in the lignin macromolecule forms benzyl ester bonds [15]. The structure has also been shown to exist in natural wood cell walls. Tanaka et al. utilized the reaction of a quinone methide of a lignin model compound with D-glucose and D-glucuronic acid in the presence of water, respectively. The findings revealed that a benzyl ester was formed by the addition of D-glucuronic acid to a quinone methide, whereas D-glucose reacted a little [16]. Meanwhile, Terashima [17] and Cathala [18] investigated the polymerization of dehydrogenated polymers (DHPs) with carboxylate-rich pectin under aqueous phase conditions using a lignin oxidase (peroxidase) and hydrogen peroxide system. It was found that a quinone methide generated during the DHP synthetic process was able to react with the carboxyl group of pectin to form a benzyl ester bond. These studies demonstrated that the benzyl ester–LCC structure is the main mode of lignin binding to hemicellulose [19]. To elucidate the formation process of LCCs in plants, Sipilä and Brunow [20,21] investigated the addition reactions of a quinone methide of lignin with vanillyl alcohol, methyl-α-D-glucopyranoside, and D-glucuronic acid in water–dioxane solutions at various pH values. The results showed that the pH value of the reaction system had an important effect on the formation of LCCs. Acidic conditions are conducive to the formation of benzyl ester bonds. The model and reaction conditions used in the above studies are very different from the formation conditions of LCCs in plants, which has a negative impact on the research results. In the authors’ previous study, the biosynthesis of the benzyl ester bonds in the LCC structure in plants was simulated by using coniferin glucoside as the lignin precursor. Under the catalysis of laccase-/β-glucosidase-/O_2_-composed lignin oxidase, the coniferin was polymerized with glucuronic acid under different pH values (pH = 4.0, pH = 5.0, pH = 6.0, pH = 7.0) to generate DHPs and DHP–glucuronic acid complexes [22]. The results of the study showed the interaction of coniferin with glucuronic acid to form DHP–glucuronic acid complexes. In addition, the pH of 4.0 favored the formation of a benzyl ester bond, which was in agreement with the above findings. Glucuronic acid is the main component of hemicellulose in softwood. However, the softwood typically comprises konjac glucomannan (KGM) in major and glucuronic acid in minor amounts.

In order to better study the formation process of benzyl ester-type LCCs between lignin and hemicellulose, the oxidation method of the TEMPO/NaClO/NaBr oxidative system was used to enrich konjac glucomannan with an appropriate amount of carboxyl groups in this work. Then, the oxidized konjac glucomannan and coniferin (the lignin precursor) were polymerized in both acidic (pH = 4.0) and neutral (pH = 7.0) conditions to produce the DHP–glucan complex. The structure of the DHP–glucan complex was studied by infrared spectroscopy and ^13^C-NMR. The results contribute to a better understanding of the formation of LCCs in plants.

## 2. Results and Discussion

### 2.1. Infrared Spectrum Analysis of DHPKGC

**Figure 1:**
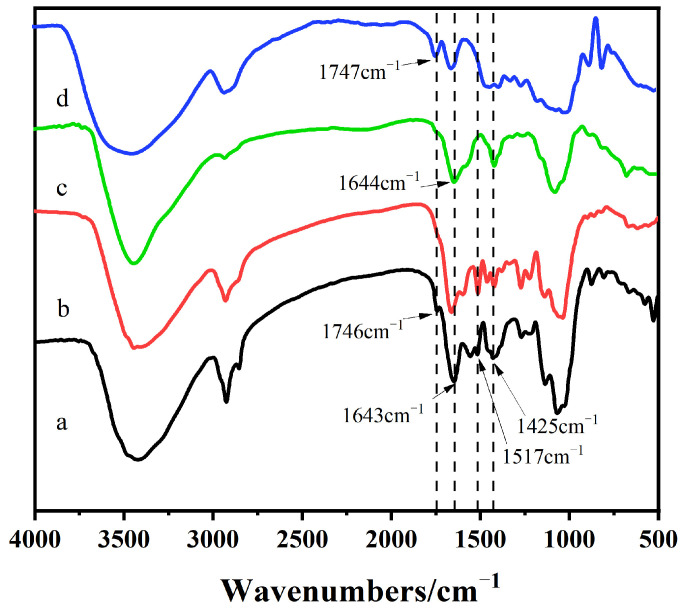
Infrared spectra of DHPCC: (a) pH = 4; (b) pH =7; (c) after TEMPO oxidation treatment; (d) original sample.

The infrared spectra of DHPKGC before and after the oxidation of KGM are shown in Figure 1. It was observed that after oxidation by the TEMPO/NaClO/NaBr oxidation system, the konjac glucomannan showed a very strong absorption peak at 1643 cm^−1^, which was the characteristic absorption peak of the carboxyl group in the polysaccharide [23]. It indicated that carboxyl groups were successfully introduced into KGM after oxidation by the oxidation system. In addition, the absorption peak of the ester bond at 1747 cm^−1^ was greatly weakened after oxidation. This is because the oxidation occurred under alkaline conditions, and the ester bond was hydrolyzed under alkaline conditions. It can be seen that the DHPKGC prepared under a pH of 4.0 appeared as a shoulder peak at 1746 cm^−1^, which was related to the absorbance peaks of carboxyl groups. However, the DHPKGC prepared at a pH of 7.0 had no absorption peak at 1746 cm^−1^. The above results showed that a pH of 4.0 was conducive to the formation of the benzyl ester bond between the carboxyl group on KGM and DHPs.

### 2.2. NMR Analysis of DHP-KGC

**Figure 2:**
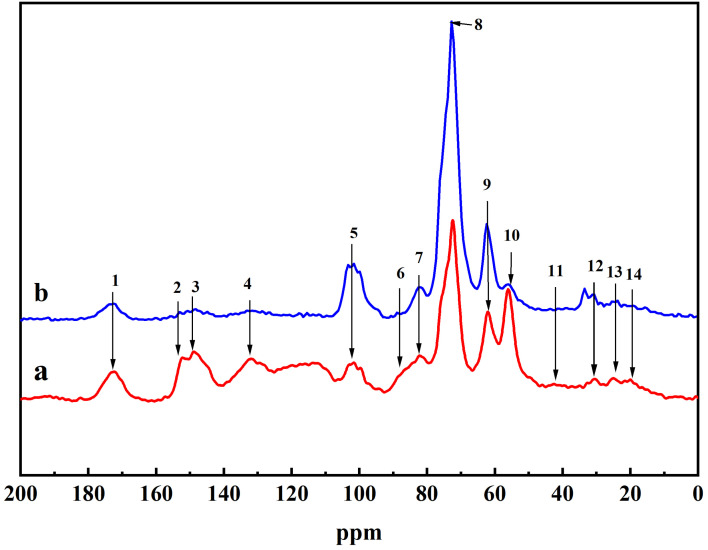
CP/MAS 13C-NMR spectra of DHPKGC prepared under pH = 7.0 (a) and pH = 4.0 (b).

The ^13^C CP/MAS NMR of the DHPKGC complex is shown in Figure 2. After the oxidation of KGM by TEMPO/NaClO/NaBr, a large number of carboxyl groups were generated on the macromolecular chain of KGM, which made the glucomannan completely dissolve in the aqueous solution. Therefore, the free KGM that was not attached to DHPs was removed during centrifugation. Since the amount of methoxy in the generated DHP was unchanged, the intensity of the absorption peak in the DHPKGC was compared using the absorption peak of methoxy as a reference. It can be seen that an intense signal at peak 5, peak 8, and peak 9 was detected, which was related to C_1_, C_2_, and C_6_ in KGM, respectively. The chemical shifts (ppm) corresponding to the structural units contained in the DHPKGC and their attributions are shown in Table 1. Significantly, the absorption peak strength of DHPKGC prepared under pH = 4.0 was stronger than that of DHPKGC prepared under pH = 7.0. At the same time, it was found that the absorption peak of the benzene ring carbon under pH = 4 was relatively low at peak 3 and peak 4. These results indicated that DHPKGC prepared under pH = 4.0 contained a large amount of KGM. The chemical shift of the benzyl ester bond in the DHPKGC at peak 8 overlapped with the C_3_ absorption peak of mannose in KGM, which made the analysis of the benzyl ester bond difficult.

### 2.3. Two-Dimensional HSQC NMR Spectrum Analysis

**Figure 3:**
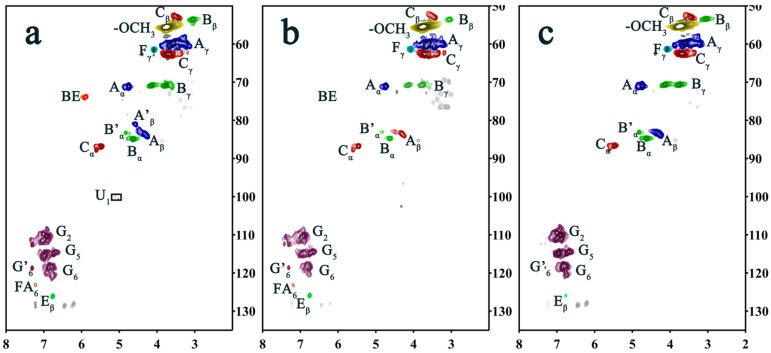
(**a**) Two-dimensional HSQC NMR spectra of EDDHPKGC prepared at pH = 4.0; (**b**) two-dimensional HSQC NMR spectra of EDDHPKGC at pH = 7.0; and (**c**) two-dimensional HSQC NMR spectra of EDDHPKGC complexes prepared at pH = 4.0 after alkali treatment.

**Figure 4:**
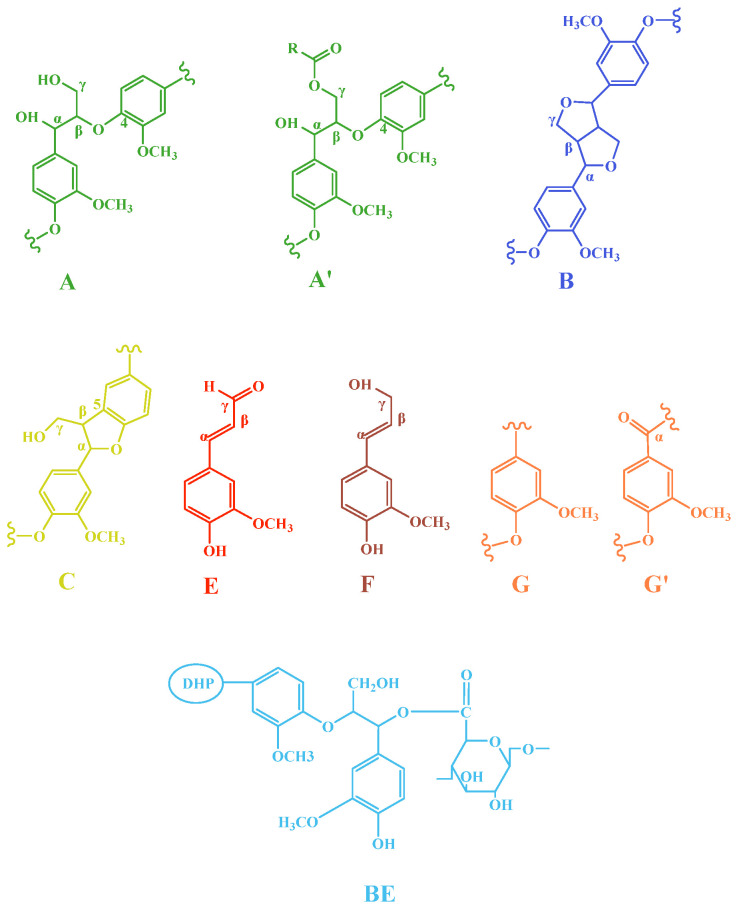
The main basic junction structure and structural unit of the side chain region and aromatic ring region in the spectrum of the DHP–cellulose complexes. A: β-O-4 ether bond structure (γ-hydroxyl); A′: β-O-4 ether bond structure (γ-acetyl group); B: resinol structure, composed of β-β and α-O-γ′; C: phenylcoumarine structure, composed of β-5 and α-O-4 connections; E: coniferaldehyde unit; F: coniferol unit; G: guaiac-based structure; G′: oxidized guaiac structure; BE: benzyl ester structure.

In order to analyze the benzyl ester bond of DHPKGC, the sample underwent enzymatic hydrolysis to further reduce the carbohydrate content, which was named EDDHPKGC. Meanwhile, the EDDHPKGC was ball-milled so that it could be dissolved in a DMSO solvent and characterized by 2D HSQC NMR. The 2D-HSQC NMR spectrum of EDDHPKGC is shown in Figure 3, and the assignments and the main basic junction structure and structural unit are presented in Table 2 and Figure 4, respectively. It was observed that the methoxyl and major lignin interunit linkages including β-O-4 aryl ether (A), phenylcoumaran (β-5) (C), and resinol (β-β) (B) were found in the aliphatic region (50–90/2.5–6.0 ppm) of the HSQC spectra. Compared with Figure 3b, it was found that a new absorption peak appeared at δ_C_/δ_H_ 73.86/5.90 ppm and 100.42/5.02 ppm in Figure 3a. The sharp signal at δ_C_/δ_H_ 73.86/5.90 ppm was attributed to the absorption peak of the benzyl ester bond. The signal at δ_C_/δ_H_ 100.42/5.02 ppm was the absorption peak of glucose and mannose (C_1_) in KGM. It is well known that the benzyl ester bond can be broken by sodium hydroxide. In order to further confirm the existence of a benzyl ester bond, the EDDHPKGC prepared under pH = 4.0 was subjected to alkaline treatment, and the 2D HSQC NMR after treatment is shown in Figure 3c. It can be seen that the absorption peaks disappeared at δ_C_/δ_H_ 73.86/5.90 ppm and δ_C_/δ_H_ 100.42/5.02 ppm, which further proves that the signal at δ_C_/δ_H_ 73.86/5.90 ppm was the absorption peak of the benzyl ester bond. As a result of the break of the benzyl ester bond, the KGM was free from the EDDHPKGC and removed in the centrifugal separation process. Hence, it can be concluded that carboxyl groups on KGM can efficiently react with quinone methides to form benzyl ester bond types for LCCs under acidic conditions, which was consistent with the results of infrared analysis.

## 3. Materials and Methods

### 3.1. Materials

Coniferin was obtained using the procedure described by Terashima and Seguchi [24]. Konjac glucomannan from konjac was provided by Hubei Yizhi Konjac Biotechnology Co., Ltd., (Yichang, China). Laccase (E.C.1.10.3.2) was purchased from Novozymes A/S (Tianjin, China). Mannanase from *Aspergillus niger* (specific activity: 10 units/mg) was obtained from YuanYe (Shanghai, China), and β-Glucosidase from almonds (specific activity: 6.3 units/mg) was purchased from Sigma-Aldrich (Shanghai, China). (2,2,6,6-tetramethylpiperidin-1-yl) oxyl (TEMPO), sodium bromide, and a 12% sodium hypochlorite solution were provided by Aladdin Industrial Corporation (Shanghai, China). All chemicals were of analytical grade and were used without further purification.

### 3.2. Methods

#### 3.2.1. Oxidation of Konjac Glucomannan

Firstly, the oxidation of konjac glucomannan was carried out by mixing konjac glucomannan (10 g), TEMPO (50 mg), and NaBr (1.0 g) with 2000 mL distilled water in a beaker. After that, the NaClO (1.5 mmol/g) was added to the beaker, and NaOH (0.1 mol/L) was added drop by drop to keep the PH at 10.5. When the reaction time was 2 h, it was adjusted to a pH of 7.0 with an HCl (0.5 mol/L) solution. After the reaction, the ethanol was poured into the reaction liquid; then, the spent liquor and solid fractions were separated through centrifugation. The surface chemical of the sample was washed with ethanol and the oxidized konjac glucomannan (OKGM) was obtained.

#### 3.2.2. Preparation of DHP–Konjac Glucomannan Complexes

The OKGM (1 g) was dissolved in phosphate buffers at pH levels of 4.0 and 7.0 (0.2 M, 50 mL), respectively. Then, laccase (1 mL) and β-glucosidase (100 mg) were added to the above solution and placed in a water bath at 30 °C. Then, the coniferin-β-D-glucoside (0.8 g) was dissolved in phosphate buffers at a pH of 4.0 and a pH of 7.0 (0.2 M, 50 mL), respectively. After that, the coniferin-β-D-glucoside solution was added to the enzyme-containing OKGM solution with a constant flow pump (flow rate of 2 mL/h). Meanwhile, the (1 mL) β-glucosidase was supplemented in the reaction system. The reaction continued for 6 days, during which air filtered through cotton and activated carbon was continuously fed. After the reaction, the precipitated part was cleaned with distilled water several times and dried in a vacuum drying oven to obtain DHP–konjac glucomannan complexes, coded as DHPKGC.

#### 3.2.3. Enzymatic Degradation of DHPKGC

Mannanase (1 g) was dissolved in a buffer solution of 0.2 M HAc/NaAc (pH = 4.0, 100 mL), and then the enzyme solution was filtered through a G3 sand-core funnel to obtain the filtered enzyme solution. The DHPKGC was added to the filtered enzyme solution and hydrolyzed in a shaking bath at 50 °C for 24 h. After the reaction, the reaction system was separated by centrifugation. In addition, the insoluble part was fully washed and dried to obtain enzyme-degraded DHPGC (coded as EDDHPKGC).

#### 3.2.4. Alkaline Treatment of EDDHPKGC

The alkaline treatment process of EDDHPKGC was carried out based on Xie et al. [25]. In detail, 100 mg of EDDHPKGC was dissolved in a NaOH solution (1 mol/L). The solution was placed under a nitrogen atmosphere and stirred at room temperature for 12 h. After that, the diluted HC1 was added to adjust the PH of the solution to acid. Finally, the precipitate was collected by centrifugation and freeze-dried.

## 4. Analytical Procedure

### 4.1. Infrared Spectroscopic Analysis of DHPKGC

Firstly, the KBr film was fabricated by mixing 1–2 mg of DHPKGC with 200 mg of anhydrous KBr. Then, infrared analysis was conducted utilizing a Thermo Fisher Nicolet (6700, Waltham, MA, USA) spectrometer.

### 4.2. CP/MAS ^13^C-NMR Spectra Analysis of DHPCC

The AV-III 400 M spectrometer (Bruker Corp., Karlsruhe, Germany) was used to observe CP/MAS ^13^C NMR pictures. The test condition employed a frequency of 100.6 MHz and an acquisition time of 0.02 s. The scan times were set aside at 3600 with a proton 90° pulse time of 3.0 s and a 3.0 s pulse delay.

### 4.3. Two-Dimensional HSQC NMR Analysis of EDDHPCC

The 2D-HSQC NMR characterization analysis was performed on a 500 MHz spectrometer (AVANCE III, Bruker, Germany). In order to fully dissolve the EDDHPCC in DMSO-d_6_, the samples were treated by ball milling. In brief, the sample was put into the planetary ball mill for 10 h. After the ball grinding was completed, the sample powder in the ball mill tank was removed for analysis. Then, 50 mg of the obtained samples and 0.5 mL of DMSO-d_6_ were placed into a 5 mL centrifuge tube. The mixture was sufficiently dissolved, and the supernatant was taken to be tested by NMR for 8 h.

## 5. Conclusions

The pH value has an important effect on the formation of benzyl ester bonds between DHPs and oxidized KGM. The acidic condition (pH = 4.0) was conducive to the occurrence of polymerization between the carboxyl group on KGM and DHPs. In addition, the methylene quinone intermediates produced during the formation of DHPs were connected to the polysaccharide in oxidized KGM mainly by the benzyl ester bond. This conclusion contributes to a better understanding of the process of LCC formation in plants.

## Figures and Tables

**Table 1:** Chemical shifts and assignments of major peaks in the CP/MAS ^13^C NMR spectra of DHPKG.

Peak No.	pH 4	pH 7	Assignments
1	172.7	173.8	-COO- in aliphatic esters and acetyl group
2	152.1	152.7	C_3_/C_4_ in G-ring
3	147.1	147.3	C_4_ in G-ring
4	131.2	131.6	C_1_ in G-ring
5	101–103	101–103	C_6_ in G-ring
6	88.3	88.6	C_α_(β-5, β-β)
7	80.5	81.2	C_β_ in β-O-4
8	74	74.3	C_α_ in β-O-4, Cα of ester
9	62.9	62.8	C_α_/C_β_ in β-1, C_γ_ in β-5, and β-O-4
10	54.7	56.9	-OCH_3_
11	42.1	42.9	α-CH_2_- side chain of phenylpropane units
12	30.9	30.4	β-CH_2_- side chain of phenylpropane units
13	24.1	24.1	-CH_2_ in saturated alkyl
14	20.7	20.1	-CH_3_ of acetyl

**Table 2:** Two-dimensional HSQC NMR spectra of EDDHPKGC.

Lable	pH = 4	pH = 4 (Alkali)	pH = 7	Assignments
	δ_C_/δ_H_ (ppm)	δ_C_/δ_H_ (ppm)	δ_C_/δ_H_ (ppm)	
C_β_	53.03/3.45	52.77/3.51	52.79/3.53	C_β_–H_β_ in phenylcoumaran (C)
B_β_	53.48/3.03	53.46/3.05	53.54/3.06	C_β_–H_β_ in β-β (resinol) (B)
OCH_3_	55.46/3.75	55.44/3.74	55.49/3.74	C–H in methoxyls
A_γ_	59.44/3.45 60.37/3.79	59.59/3.40 59.87/3.77	59.74/3.34 60.32/3.75	C_γ_–H_γ_ in β-O-4 substructures (A)
F_γ_	61.39/4.08	61.45/4.08	61.46/4.08	C_γ_–H_γ_ in cinnamyl alcohol end groups (F)
C_γ_	62.64/3.72	62.57/3.71	62.59/3.72	C_γ_–H_γ_ in phenylcoumaran (C)
B_γ_	70.86/3.75 70.81/4.13	70.77/3.74 70.81/4.13	70.74/3.74 70.83/4.13	C_γ_–H_γ_ in β-β resinol (B)
A_α_	71.05/4.74	71.06/4.74	70.13/4.73	C_α_–H_α_ in β-O-4 unit (A)
BE	73.86/5.90	ND	ND	Benzyl ester bond structure (BE)
A_β_	83.99/4.33	83.66/4.28	83.58/4.28	C_β_–H_β_ in β-O-4 substructures (A)
A′_β_	80.96/4.56	ND	ND	C_β_–H_β_ in β-O-4 linked to G (A)
B_α_	84.83/4.62	84.85/4.62	84.90/4.63	C_α_–H_α_ in β-β resinol (B)
B′_α_	83.21/4.83	83.16/4.83	83.15/4.83	C_α_–H_α_ in β-β (B′, tetrahydrofuran)
C_α_	86.79/5.49 87.69/5.60	86.77/5.47 87.86/5.63	86.84/5.46 87.75/5.59	C_α_–H_α_ in phenylcoumaran (C)
G_2_	108.58/6.90 111.46/7.04 112.50/7.42	110.13/6.91 111.50/7.06 112.33/7.42	110.35/6.91 112.57/7.57	C_2_–H_2_ in guaiacyl units (G)
G_5_	114.80/6.73 114.92/6.95	114.49/6.71 114.89/6.95	114.55/6.75 115.13/6.96	C_5_–H_5_ in guaiacyl units (G)
G_6_	118.31/6.85 120.30/6.75	118.50/6.84	118.63/6.76	C_6_–H_6_ in guaiacyl units (G)
G′_6_	118.70/7.31	118.95/7.33	118.76/7.31	α C_6_–H_6_ in G-type structural units with oxidized sites
E_β_	126.04/6.76	126.06/6.76	126.06/6.75	C_β_–H_β_ in cinnamyl aldehyde end groups (E)
U_1_	99.82/5.11 100.42/5.02	ND	ND	C_1_–H_1_ in 4-O-methyl-α-D-GlcUA (U)
FA_6_	123.03/7.22	ND	123.41/7.18	C_6_–H_6_ in ferulate (p-FA)

## Data Availability

Data is contained within the article.

## References

[1] Pang Y.X., Sharmin N., Wu T., Pang C.H. (2023). An investigation on plant cell walls during biomass pyrolysis: A histochemical perspective on engineering applications. Appl. Energy.

[2] Riseh R.S., Fathi F., Lagzian A., Vatankhah M., Kennedy J.F. (2024). Modifying lignin: A promising strategy for plant disease control. Int. J. Biol. Macromol..

[3] Sarkanen K.V., Ludwig C.H. (1971). Lignins: Occurrence, Formation, Structure and Reactions.

[4] Björkman A. (1957). Lignin and lignin–carbohydrate complexes–extraction from wood meal with neutral solvents. Ind. Eng. Chem..

[5] Zhao Y., Shakeel U., Rehman M.S.U., Li H., Xu X., Xu J. (2020). Lignin-carbohydrate complexes (LCCs) and its role in biorefinery. J. Clean. Prod..

[6] Giummarella N., Pu Y., Ragauskas A.J., Lawoko M. (2019). A critical review on the analysis of lignin carbohydrate bonds. Green Chem..

[7] Akpan E.I., Wetzel B., Friedrich K. (2021). Eco-friendly and sustainable processing of wood-based materials. Green Chem..

[8] Liu C., Luan P., Li Q., Cheng Z., Xiang P., Liu D., Hou Y., Yang Y., Zhu H. (2021). Biopolymers derived from trees as sustainable multifunctional materials: A review. Adv. Mater..

[9] Mäkelä M.R., Hildén K., Kowalczyk J.E., Hatakka A. (2020). Progress and Research Needs of Plant Biomass Degradation by Basidiomycete Fungi. Grand Challenges in Fungal Biotechnology.

[10] Liu Y., Wang X., Wu Q., Pei W., Teo M.J., Chen Z.S., Huang C. (2022). Application of lignin and lignin-based composites in different tissue engineering fields. Int. J. Biol. Macromol..

[11] Zhao H., Li J., Wang P., Zeng S., Xie Y. (2017). Lignin-Carbohydrate Complexes Based Spherical Biocarriers: Preparation, Characterization, and Biocompatibility. Int. J. Polym. Sci..

[12] Zhao H., Feng Q., Xie Y., Li J., Chen X. (2017). Preparation of biocompatible hydrogel from lignin-carbohydrate complex (LCC) as cell carriers. BioResources.

[13] Yaku F., Yamada Y., Koshijima T. (1976). Lignin carbohydrate complex Pt. II. Enzymic degradation of acidic polysaccharide in Björkman LCC. Holzforschung.

[14] McKendry P. (2002). Energy production from biomass (part 1): Overview of biomass. Bioresour. Technol..

[15] Sipilä J., Brunow G. (1991). On the mechanism of formation of non-cyclic benzyl ethers during lignin biosynthesis. Part 4. The reactions of a β-O-4-type quinone methide with carboxylic acids in the presence of phenols. The formation and stability of benzyl esters between lignin and carbohydrates. Holzforschung.

[16] Tanaka K., Nakatsubo F., Higuchi T. (1979). Reactions of guaiacylglycerol-beta-guaiacyl ether with several sugars. II. Reactions of quinonemethide with pyranohexoses. J. Wood Sci..

[17] Terashima N., Atalla R., Ralph S., Landucci L.L., Lapierre C., Monties B. (1995). New preparations of lignin polymer models under conditions that approximate cell wall lignification. I. Synthesis of novel lignin polymer models and their structural characterization by ^13^C NMR. Holzforschung.

[18] Cathala B., Monties B. (2001). Influence of pectins on the solubility and the molar mass distribution of dehydrogenative polymers (DHPs, lignin model compounds). Int. J. Biol. Macromol..

[19] Freudenberg K., Grion G. (1959). Beitrag zum Bildungsmechanismus des Lignins und der Lignin-Kohlenhydrat-Bindung. Chem. Ber..

[20] Brunow G., Sipilä J., Mäkelä T. (1989). On the Mechanism of Formation of Non-cyclic Benzyl Ethers During Lignin Biosynthesis. Part 1. The Reactivity of β-O-4 Quinone Methides with Phenols and Alcohols. Holzforschung.

[21] Sipilä J., Brunow G. (1991). On the Mechanism of Formation of Non-Cyclic Benzyl Ethers During Lignin Biosynthesis. Part 2. The Effect of pH on the Reaction between a β-O-4-Type Quinone Methide and Vanillyl Alcohol in Water-Dioxane Solutions. The Stability of Non-Cyclic Benzyl Aryl Ethers During Lignin Biosynthesis. Holzforschung.

[22] Wang X., Le X., Peng W., Ye P., An J., Zhang G., Wang P., Xie Y. (2022). Effect of pH on the formation of benzyl ester bonds between glucuronic acid and dehydrogenation polymer. BioResources.

[23] Bu X., Pei J., Zhang F., Liu H., Zhou Z., Zhen X., Wang J., Zhang X., Chan H. (2018). The hydration mechanism and hydrogen bonding structure of 6-carboxylate chitooligosaccharides superabsorbent material prepared by laccase/TEMPO oxidation system. Carbohydr. Polym..

[24] Xie Y., Terashima N. (1994). Selective carbon 13-enrichment of side chain carbons of ginkgo lignin traced by carbon 13 nuclear magnetic resonance. J. Jpn. Wood Res. Soc..

[25] Xie Y., Yasuda S., Wu H., Liu H. (2000). Analysis of the structure of lignin-carbohydrate complexes by the specific ^13^C tracer method. J. Wood Sci..

